# Incentive-compatible mechanism for manufacturing carbon emission supervision under carbon control policies in China

**DOI:** 10.1371/journal.pone.0299086

**Published:** 2024-05-13

**Authors:** Peipei Liang, Youqing Lv, Yajuan Zhao

**Affiliations:** School of Economics and Management, Anqing Normal University, Anqing, China; Harbin Institute of Technology, CHINA

## Abstract

Enhance performance in manufacturing carbon emission (MCE) reduction has become a widespread consensus and a necessary part, which cannot be achieved without the joint participation of manufacturing enterprises and supervisory departments. Accordingly, how to coordinate the interests of both sides and design a reasonable incentive-compatible mechanism becomes an urgent task at present. Considering the two subsidy funding channels of peer funds and government finance, this study applies the evolutionary game model to analyze feasible schemes for designing incentive-compatible mechanism of MCE supervision, discusses and simulates the realistic scenarios and influencing factors of incentive-compatible mechanism under the non-subsidized and subsidized schemes. The results show that MCE supervision is in an incentive-incompatible state under the non-subsidized incentive scheme, while in a constrained incentive-compatible state under the subsidized incentive scheme. With the increase of peer funds and penalty coefficient or the decrease of subsidy coefficient, the period of MCE supervision to reach an incentive-compatible state becomes shorter. However, a lower peer fund and penalty coefficient or a higher subsidy coefficient will contribute to a state of incentive-incompatible or a periodic cycle state of "incentive-compatible → incentive-incompatible →incentive-compatible→…" in the MCE supervision.

## Introduction

Against the backdrop of global climate change, carbon emission reduction (CER) has become a global goal and consensus [1]. As the world’s largest developing country and the major emitter of greenhouse gases, China has adopted policies such as carbon tax, carbon cap-and-trade and carbon offset to achieve the goals of “carbon peaking” by 2030 and “carbon neutrality” by 2060. Manufacturing is the second largest source of carbon emissions in China behind the energy generation industry [2], and its ability to reduce carbon emissions is crucial to green manufacturing transition [3]. Stimulated by a series of carbon policies such as carbon taxes and penalties, China’s manufacturing industry has achieved remarkable results in CER since 2016. However, the share of manufacturing value-added in GDP has continued to decline over the same period, reflecting the dilemma of not being able to achieve both economic gains and carbon reduction [4].

For enterprises, the introduction of carbon trading encourages profit-seeking firms to regulate emissions by incentivizing efficiency increase and emission curbing [5]. However, due to the negative externalities of carbon over-emission behavior associated with marginal abatement cost [6], MCE has repeatedly violated, and even recklessly falsified inspection reports and made false coal samples. According to statistics from China emission license management information platform, in the past three years, 35 out of 170 third-party verification service agencies issued unqualified verification conclusions, involving 148 manufacturing enterprises.

As a matter of fact, the nature of MCE violations under the carbon control scenario is a problem of incentive-incompatible between supervisory objectives and supervisory policies. The main reason is that in the supervision process of carbon emission in China, there is a high degree of information asymmetry between supervisory department and manufacturing enterprise, which inevitably generates a conflict of objectives. According to the practical application of incentive regulation theory [7, 8], the key to eliminate the information asymmetry problem in supervision is to achieve incentive compatibility between the supervisor and supervised, and to moderate potential profit conflicts. To our knowledge, the Chinese government mainly adopts a penalty-based incentive mechanism in MCE supervision, and incentive mechanisms such as green subsidies and carbon offsets are relatively narrow in coverage. However, Fehr et al. [9] and Herrmann et al. [10] argue that penalty does not have economic effects and may even lead to socially retaliatory behavior. Therefore, based on the concept of incentive-compatible, this study attempts to analyze the incentive-incompatible problem in current Chinese MCE supervision and discusses how to establish an incentive-compatible carbon emission supervision mechanism with the purpose of providing a new perspective for carbon emission supervision supervisory departments to expand their supervisory thinking and improve supervisory efficiency. The main problems to be solved in this paper are as follows:

1. What are the evolution characteristics of the game system between manufacturing enterprises and local governments under different reward and penalty modes?
2. Which reward and penalty model implemented by local governments under the supervision of MCE has the best effect?
3. How does reward and penalty affect the evolution of behavioral strategies of manufacturing enterprises and local governments?

Compared with the former studies, the main contributions of this study include: (1) Taking incentive-incompatible as the breakthrough point, we explore the opportunistic behaviors and profit conflicts among different subjects due to information asymmetry. Through normative research, the lack of supervision constraint and efficiency enhancement of MCE is remedied. (2) Considering the problem of government financial pressure, an incentive-compatible mechanism containing peer funds and official subsidies is designed, and different scenarios and establishment conditions of interactive behaviors between manufacturing enterprises and supervisory departments are theoretically summarized. (3) Against the special background of dual-carbon policy, the study of MCE supervision has a certain timeliness, which can provide theoretical support for the government to form the main measure and institutional arrangement of an incentive-compatible supervision mechanism.

The rest of this study is organized as follows: The Literature review section presents a summary of the relevant former studies; while the Methodology section constructs game models for the behavioral evolution of manufacturing enterprises and supervisory departments under non-subsidized and subsidized incentives, and analyzes the MCE incentive compatibility scenarios. The Case and simulation analysis section performs numerical simulations of the evolutionary trajectories of the game system and the incentive effects of different factors under non-subsidized and subsidized incentives. The main conclusions are provided in the Conclusions section, along with recommendations for future research.

## Literature review

### CER

CER is the key to overcoming global resource constraints and achieving sustainable development. With regard to CER topic, scholars have mainly focused on assessing CER potential [11, 12], implementing CER measures [13, 14] and evaluating CER efficiency [15, 16]. Generally, at the firm level, CER measures fall into two broad categories, namely technology and operation, and should consider the actual CER pressure that the region or enterprise can afford, i.e., CER potential. CER efficiency is a commonly used indicator when assessing CER potential [17], based on various dimensions such as energy efficiency [18, 19], renewable energy [20], technology [21–23], and operations management [24, 25]. Additionally, some scholars have studied the impact of financing capacity and effective governance on CER efficiency [26, 27]. Bhandari et al. [28] argued that energy efficiency gaps due to financial and market factors, hinder the immediate spending in energy saving technologies. An et al. [29] focused on the firms’ willingness to pay for CER, and found that the characteristics of an individual firm, such as profitability, production scales, responsibility, etc., positively affect the willingness on carbon abatement.

### Carbon emission supervision

After the Great Depression in the 1920s to 1930s, supervision proved to be an effective means for governments to address market abuses (e.g., fraud, or conflicts of profit), and the rationale for strengthening government supervision gained wider social acceptance [30]. Wang [31] defined government supervision as a variety of legal constraints and incentives that government administrative agencies with supervisory functions take on micro market actors based on public interest objectives. Usually, government supervision can be divided into two types: command-and-control type and market-incentive type. The former is related to environmental regulation clauses, pollutant discharge standards, and cleaner production standards [32, 33]. The latter category includes carbon emissions trading, carbon tax (pollution charges), and subsidies [34, 35]. In actuality, the themes of carbon disclosure [36, 37], opportunism [38], corruption [39] and legal criminal liability [40, 41] have directly or indirectly validated the necessity of government supervision mechanisms. Furthermore, Meng et al. [42] and Jiang et al. [43] verified the effective influence of government supervision on CER, while also suggesting that it may lead to a prisoner’s dilemma for manufacturing firms. Thus, designing appropriate incentive mechanisms under different levels of government supervision is important and urgent in improving carbon efficiency and promoting green transition.

### Incentive-compatible mechanism

Given the principal-agent relationship prevalent in society [44], agents usually have more information than principals, which generates an incentive problem. However, the agent’s information superiority situation leads to efficient, fair, and incentive-compatible properties that are prone to be in conflict [45], making early research results argue for the fundamental impossibility of mechanism design [46, 47]. With the development of research techniques and methods, scholars have made more attempts in resolving the aforementioned incentive conflicts. David [48] and Xie et al. [49] designed incentive-compatible mechanisms based on a financial contract difference and market-based coordination methods, respectively. Enaleev [50] argued that optimal mechanism design should include planning procedure, penalty and incentive functions, while Mansour et al. [51] developed an incentive-compatible bandit algorithm. These mechanisms have played an important value in ensuring incentive effectiveness and economic efficiency, and are widely used in research areas such as reactive power resource provisions [52], clean-development investments [53], and public goods provisions [54].

Moreover, as an effective tool to study the impact of institutional evolution [55, 56], the evolutionary game can effectively portray the problem of profit claims and conflicts among multiple subjects. Accordingly, scholars used the evolutionary game to explore the main reasons of incentive-incompatible among economic subjects and form the institutional arrangement of incentive-compatible mechanism. For instance, Sun et al. [7] analyzed the behavioral motives and profit demands of governments, enterprises and public in the context of low-carbon economic development and found the breakthrough point for formulating relevant incentive-compatible policies. To explore incentive-compatible payment mechanisms for watershed services, Sheng and Webber [57] and Jiang et al. [8] validated the effect of the supervisor’s penalty and compensation intensity on incentive-compatible schemes through a tripartite evolutionary game model. While Sheng et al. [58] explored the factors influencing the strategies of each stakeholder in environmental regulation, and equally tested incentive-compatible regulatory policies through the evolutionary game.

The above studies have laid a certain theoretical foundation for the design of incentive-compatible mechanism for carbon emission supervision, but there are still the following shortcomings. First, some studies examined the utility or the impacts of environmental regulation on enterprises’ competitiveness, innovation and productivity. However, the enterprise-to-government conflicts of interest have received little attention. Especially in the case of China’s regime, characterized by a combination of political concentration and economic decentralization, it is necessary to develop a deeper understanding of the incentive-compatible between the supervisors and supervised. Second, the optimization of enterprises’ own financing channels can alleviate the pressure of financial payments and indirectly promote the implementation of subsidy policies, but few scholars have studied the funding channels of subsidy policies. Existing studies about the reward-penalty incentive mechanism are usually based on subsidy policies with a single official funding channel, and few studies have been conducted on incentives based on multiple funding channels. Therefore, the application of evolutionary game theory and implementation of incentive-compatible carbon emission supervision based on multiple funding channels, can provide a new direction for tackling the profit conflict between different participants in China’s normative carbon emission.

## Methodology

### Game situation and assumptions

Since the official launch of China’s carbon market on-line trading in July 2021, the development of the supervisory departments has shown a scale effect in the MCE market. In the reality of information asymmetry, there are different groups of behavioral strategies in both manufacturing enterprises and supervisory departments. Specifically, the behavioral strategies of manufacturing enterprises can be divided into "compliant emission" and "non-compliant emission". "compliant emission" refers to the behaviors of proactively addressing social responsibilities, reasonably formulating CER plans, regularizing CER activities and legally disclosing information in accordance with carbon quotas and emission reduction commitments, while "non-compliant emission" refers to the behaviors of violating emission standards, emitting in excess of quota allowances, not completing carbon compliance within the required timeframe, misreporting and underreporting greenhouse gas emissions, etc. Accordingly, local supervisory departments, as the specific subjects of environmental regulation policy implementation, face "dual governance" from central ministry of environmental protection and local governments in China. Local governments, for the sake of economic growth performance, require local supervisory departments to relax environmental regulation. Local supervisory departments are forced to cooperate with local government decisions for the sake of political promotion and department interest, which may deviate from the central government’s goals in regulating carbon emissions [59]. In view of this, supervisory departments can choose either "strict supervision" or "relaxed supervision" strategy.

**Table 1** lists the main symbols and their meanings involved in this study.

**Table 1:** The symbols and their meanings.

Symbol	Description
*r*	The normal return obtained by manufacturing enterprises
*α*	The degree of non-compliant emission
*c*	The cost of non-compliant emission
*αc*	The cost of compliant emission
*θ*	The penalty coefficient imposed by supervisory departments
*λ*	The degree of relaxed supervision
*k*	The supervision cost of strict supervision
*λk*	The supervision cost of relaxed supervision
*w*	The return obtained by supervisory departments under "strict supervision" strategy
*p*	The probability of being detected under " relaxed supervision" strategy
*v*	The peer fund deposited by manufacturing enterprises
*β*	The subsidy coefficient imposed by supervisory departments
*x*	The probability of "compliant emission" strategy
*y*	The probability of "strict supervision" strategy

Assumption 1: Consistent with the fundamental nature of the evolutionary game [60], both manufacturing enterprises and supervisory departments are finite rational. In the repeated game, when supervisory departments adopt “strict supervision” strategy, manufacturing enterprises’ violations will be promptly detected and placed in administrative penalty.

Assumption 2: The probability of manufacturing enterprises choosing the "compliant emission" and "non-compliant emission" strategies are *x* and 1 ‒ *x*, respectively, and the normal return under both strategies is *r*. Drawing on the research hypothesis of Wei et al. [61], the manufacturing enterprises’ degree of non-compliant is *α*. Then, the cost of non-compliant emission and compliant emission are *c* and *αc*, respectively, which means that the manufacturing enterprises’ additional return of non-compliant emission is (*α* ‒ 1)*c*. Correspondingly, the potential penalty for manufacturing enterprises’ non-compliant emission is *θα*^2^(*α* ‒ 1)*c*. Among them, *θ* is the penalty coefficient, which indicates the supervisory department’s penalty intensity.

Assumption 3: The probability of supervisory departments choosing the "strict supervision" and "relaxed supervision" strategies are *y* and 1 ‒ *y*, respectively. When the supervisory department strictly supervises, the supervision cost is *k* and the return on performance that can be obtained is *w*. Conversely, when the supervisory department’s loose degree of supervision on manufacturing enterprises is *λ*, the supervision cost is *λk*. Considering the contingent nature of risk occurrence [62, 63], the probability of manufacturing enterprises being "named by the environmental verification agency" or exposed in the media for non-compliance is *p* under the "relaxed supervision" strategy of supervisory departments. At this point, the supervisory department’s performance return will be (1 − *p*)*w*.

To make the above assumptions more realistic, the conditions of 0 ≤ {*x*,*y*,*p*} ≤ 1,*p* > 0,*α* > 1,0 < *λ* < 1 and *θ* > 0 are constantly satisfied.

### Model construction and analysis under non-subsidized incentive mechanism (Model I)

According to the above assumptions, the payment matrix of the game between manufacturing enterprises and supervisory departments is shown in Table 2.

**Table 2:** The game payment matrix for Model I assumption scenario.

Players		Manufacturing enterprises	
		compliant emission	non-compliant emission
**Supervisory departments**	strict supervision	*r* − *ac*,	*r* − *c*-*θα*^2^(*α* − 1)*c*,
		*w* − *k*	*w* + *θα*^2^(*α* − 1)*c* − *k*
	relaxed supervision	*r*–*ac*,	*r*–*c*,
		*w*– *λk*	(1 –*p*) *w*– *λk*

Based on the Malthusian dynamic equation theorem, the expected returns of different strategies can be obtained, seen in the **S1 Appendix**.

Thus, the replication dynamic equations of manufacturing enterprises and supervisory departments are shown in Formula (1) and (2).


$$F(x)=x(1-x)\left(\theta \alpha^{2}y-1\right)(\alpha -1)c$$ (1)

$$F(y)=y(1-y)\left\{\theta \alpha^{2}(a-1)c+pw+(\lambda -1)k-\left[pw+\theta \alpha^{2}(a-1)c\right]x\right.$$ (2)

Let Formula (1), *F*(*x*) = 0, and Formula (2), *F*(*y*) = 0, then we can obtain four fixed local equilibrium points that always exist in the game system of manufacturing enterprises and supervisory departments, namely, *E*_1_(0,0), *E*_2_(1,0), *E*_3_(0,1) and *E*_4_(1,1). Meanwhile, the partial derivatives of *F*(*x*), *F*(*y*) with respect to x, y are solved to obtain the Jacobi matrix *J*(*x*,*y*).


$$\begin{array}{l} J(x,y)=\left[\begin{array}{c} \frac{\partial U(x)}{\partial x}\;\frac{\partial U(x)}{\partial y}\\ \frac{\partial U(y)}{\partial x}\;\frac{\partial U(y)}{\partial y} \end{array}\right]\\ =\left[\begin{array}{cc} (1-2x)\left(\theta \alpha^{2}y-1\right)(\alpha -1)c & x(1-x)\theta \alpha^{2}(\alpha -1)c\\ -y(1-y)\left[pw+\theta \alpha^{2}(\alpha -1)c\right] & (1-2y)\left\{\begin{array}{c} \theta \alpha^{2}(\alpha -1)c+pw+(\lambda -1)k\\ -x\left[pw+\theta \alpha^{2}(\alpha -1)c\right] \end{array}\right. \end{array}\right] \end{array}$$ (3)

When the Jacobian matrix has determinant $Det(J)=\frac{\partial U(x)}{\partial x}*\frac{\partial U(y)}{\partial y}-\frac{\partial U(x)}{\partial y}*\frac{\partial U(y)}{\partial x}>0$ and trace $Tr(J)=\frac{\partial U(x)}{\partial x}+\frac{\partial U(y)}{\partial y}<0$ the equilibrium point is the evolutionary stability strategy (ESS) [64]. Therefore, taking *E*_4_(1,1) as an example, it can be calculated as:

$$J(1,1)=\left[\begin{array}{cc} -\left(\theta \alpha^{2}-1\right)(\alpha -1)c & 0\\ 0 & -(\lambda -1)k \end{array}\right]$$

Because of the existence of *α* >1,0 < *λ* < 1 and *θ* > 0, *J*(1,1) cannot satisfy the evolutionary stability conditions of both *Det*(*J*) > 0 and *Tr*(*J*) < 0, meaning that the equilibrium point *E*_4_(1,1) is not an ESS. Analogously, *E*_2_(1,0) also fails to evolve into a stable equilibrium, while the stability of *E*_1_(0,0) and *E*_3_(0,1) depends on the following two scenarios.

**Fig 1:**
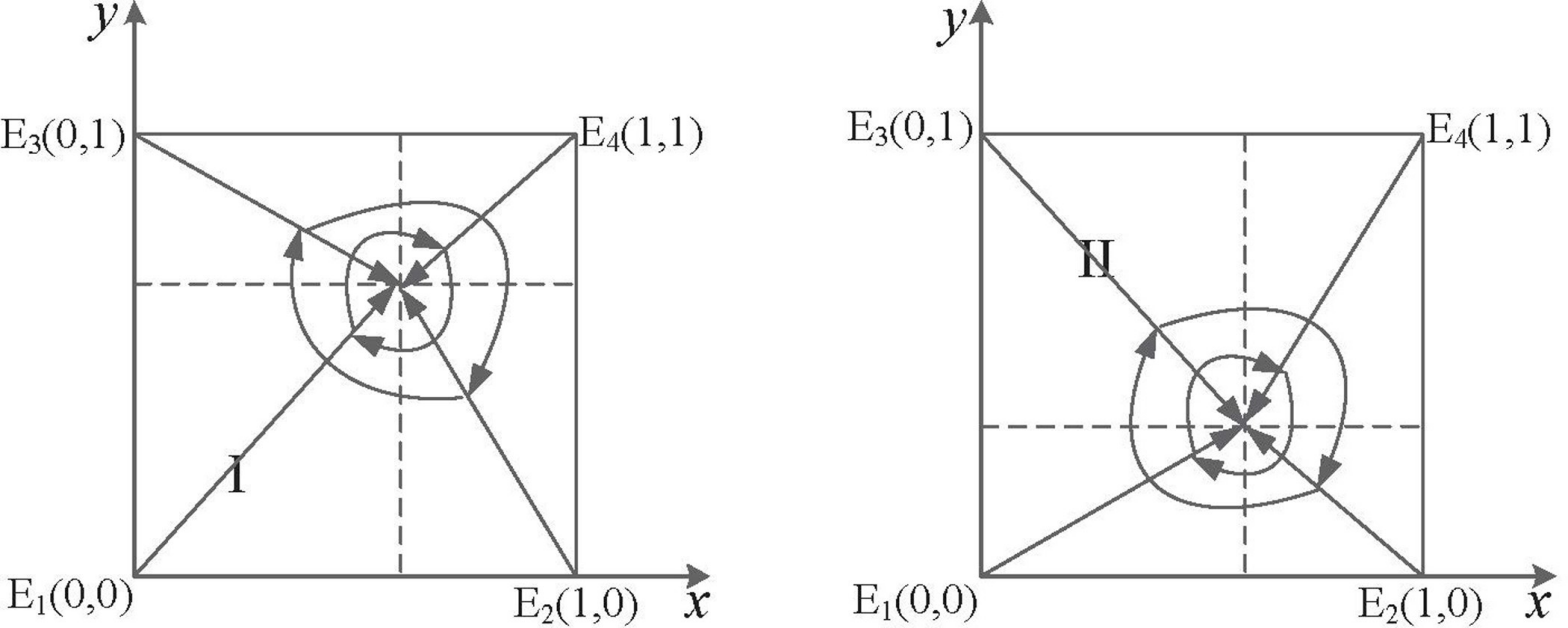
Dynamic evolution under different scenarios. (a) Scenario I. (b) Scenario II.

Scenario I: When *θα*^2^(*α* − 1)*c* + *pw* + (*λ* − 1)*k* < 0, that is, in the case of the administrative penalty of "strict supervision" is less than the additional return of "relaxed supervision", supervisory departments choose the "relaxed supervision" strategy, and manufacturing enterprises choose the "non-compliant emission" strategy, corresponding to the evolutionary game trajectory shown in Fig 1(A). Eventually, *E*_1_(0,0)evolves to ESS.

Scenario II: When *θα*^2^(*α* − 1)*c* + *pw* + (*λ* − 1)*k* > 0 and *θα*^2^ < 1, that is, in the case of the administrative penalty of "strict supervision" is more than the additional return of "relaxed supervision" and the penalty coefficient is less than a specific threshold, supervisory departments choose the "strict supervision" strategy, and manufacturing enterprises choose the "non-compliant emission" strategy, corresponding to the evolutionary game trajectory shown in Fig 1(B). As a result, *E*_2_(0,1) evolves to ESS.

From the above analysis, it can be seen that under the supervision mechanism with only pure penalty, it is difficult for the manufacturing enterprises’ strategies to evolve to "compliant emission" eventually, no matter what strategies supervisory departments choose, which is against the original purpose of market supervision by the regulators and is not conducive to the effective regulation of non-compliant carbon emission behaviors.

### Model construction and analysis under subsidized incentive mechanism (Model II)

To address the above dilemma, this study designs an incentive mechanism with positive subsidy and negative penalty based on the current pure penalty incentive mechanism. However, conventional government subsidy fund is derived from the fiscal budget, which, during longer subsidy cycles, often results in greater financial pressure on governments. Extremely, it can easily lead to the problem of incentive failure such as subsidy funding default or suspension. For this reason, this study modifies the single-channel financial incentive into a dual-channel peer incentive, that is, all manufacturing enterprises finance a peer incentive fund pool to obtain government-licensed carbon verification qualification and entrust the government supervisory departments to coordinate the management of the peer fund. When the verification quality fulfills the official standards, the supervisory departments subsidize a certain percentage of the financial subsidy and the peer fund. Among them, the financial subsidy fund is originated from the performance return of CER. Based on assumptions 1–3 in Model I, the following additional assumption is given.

Assumption 4: Referring to the incentive mechanism designed by Lu et al. [65], under the premise of "strict supervision" strategy, the supervisory departments will subsidize the compliance behavior of manufacturing enterprises and the subsidy expenditure is *β*(*w* + *v*). Among them, *v* is the peer fund deposited by the manufacturing enterprises, and β is the subsidy coefficient of the supervisory departments.

Based on the assumptions of Model II, the payment matrix of the game system is shown in Table 3.

**Table 3:** The game payment matrix for Model II assumption scenario.

Players		Manufacturing enterprises	
		compliant emission	non-compliant emission
**Supervisory departments**	strict supervision	*r* + *β*(*w* +*v*) − *αc*–*v*,	*r* − *c* − *v*–*θα*^2^(*α* − *1*)*c*,
		*w* + *v* − *k*–*β*(*w* + *v*)	*w* + *v* + *θα*^2^(*α* − *1*)*c* − *k*
	relaxed supervision	*r* − *αc*,	*r*–*c*,
		*w*– *λk*	(1 –*p*) *w*– *λk*

Correspondingly, the game model II for manufacturing enterprises and supervisory departments can be calculated.


$$\left\{\begin{array}{l} F(x)=x(1-x)\left\{(1-\alpha )c+\left[\beta (w+v)+\theta \alpha^{2}(\alpha -1)c\right]y\right\}\\ F(y)=y(1-y)\left\{v+\theta \alpha^{2}(\alpha -1)c+pw+(\lambda -1)k-\left[pw+\beta (w+v)+\theta \alpha^{2}(\alpha -1)c\right]x\right\} \end{array}\right.$$ (4)

The Jacobi matrix of the game system in mode II is obtained from Formula (4).


$$J(x,y)=\left[\begin{array}{cc} (1-2x)\left\{(1-\alpha )c+\left[\beta (w+v)+\theta \alpha^{2}(\alpha -1)c\right]y\right\} & x(1-x)\left[\beta (w+v)+\theta \alpha^{2}(\alpha -1)c\right]\\ -y(1-y)\left[pw+\beta (w+v)+\theta \alpha^{2}(\alpha -1)c\right]\; & (1-2y)\{\begin{array}{c} v+\theta \alpha^{2}(\alpha -1)c+pw+(\lambda -1)k\\ -x\left[pw+\beta (w+v)+\theta \alpha^{2}(\alpha -1)c\right] \end{array} \end{array}\right]$$ (5)

As the analysis of Model I shows, when *E*_1_(0,0) and *E*_3_(0,1) evolve to the stable equilibrium point, the behavioral strategies of the manufacturing enterprises fail to satisfy the supervisory expectation, therefore, only the evolutionary stability of *E*_2_(1,0) and *E*_4_(1,1) are discussed further below. Substituting *E*_2_(1,0) and *E*_4_(1,1) into Formula (5), we can get Formulas (6) and (7), respectively.


$$J(1,0)=\left[\begin{array}{cc} -(1-\alpha )c & 0\\ 0 & v+\theta \alpha^{2}(\alpha -1)c+pw+(\lambda -1)k \end{array}\right]$$ (6)

$$J(1,1)=\left[\begin{array}{cc} -\left[(1-\alpha )c+\beta (w+v)+\theta \alpha^{2}(\alpha -1)c\right] & 0\\ 0 & -[v+(\lambda -1)k-\beta (w+v) \end{array}\right]$$ (7)

Because of 1 –*α* < 0, *E*_2_(1,0) cannot evolve to ESS. And when *E*_4_(1,1) evolves to ESS, there exists the following constant condition.


$$\left\{\begin{array}{c} (1-\alpha )c+\beta (w+v)+\theta \alpha^{2}(\alpha -1)c>0\\ v+(\lambda -1)k-\beta (w+v)>0 \end{array}\right.$$ (8)

By further solving Formula (8), the conditional scenario for the evolution of *E*_4_(1,1) is obtained.

**Fig 2:**
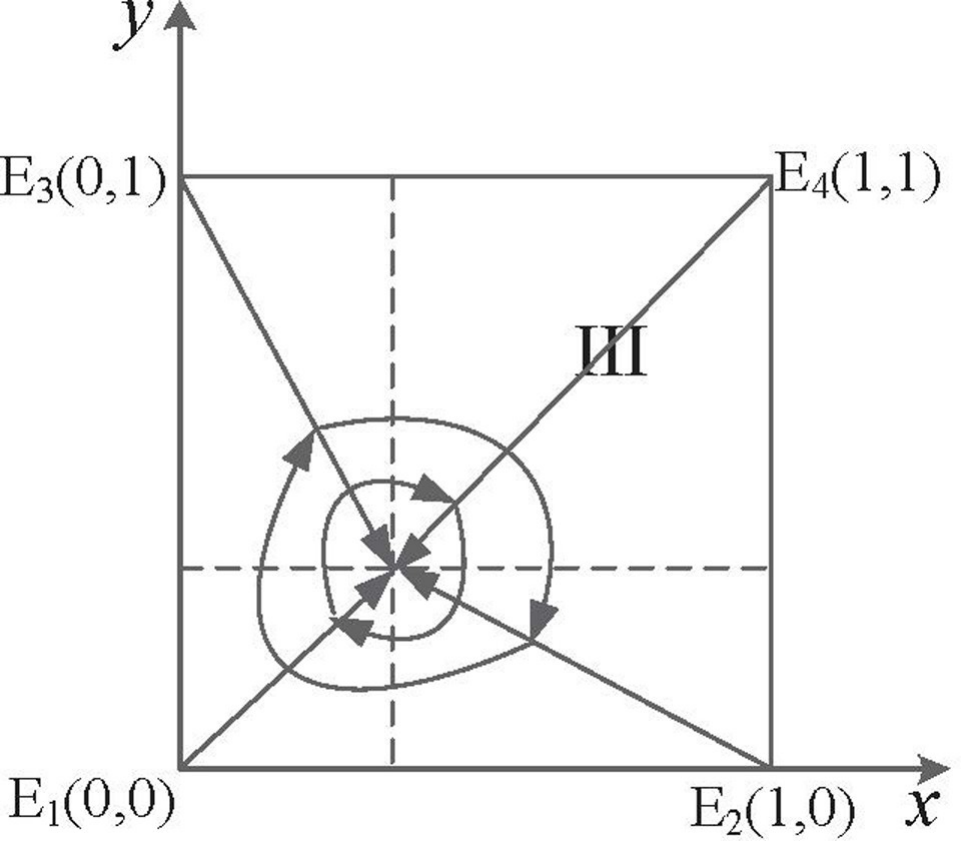
Dynamic evolution of the game system under scenario III.

Scenario III: When $\frac{\left(1-\theta \alpha^{2}\right)(\alpha -1)c}{w+v}<\beta <\frac{v+(\lambda -1)k}{w+v}$, that is, in the case of the subsidy coefficient of "strict supervision" is in a specific critical range, supervisory departments choose the "strict supervision" strategy, and manufacturing enterprises choose the "compliant emission" strategy, corresponding to the evolutionary game trajectory shown in Fig 2. Here, *E*_4_(1,1) evolves to ESS.

## Case and simulation analysis

### Computational experimental simulation

According to the research of Fang et al. [66], under the current carbon trading market mechanism in China, the cost of carbon emission reduction of enterprises is about 60 yuan/ton *CO*_2_, and the input of enterprise’s non-compliant emission should be reduced by about 30% compared with that in the case of compliant emission, so the initial value of the cost of enterprise’s non-compliant emission is set to *c* = 40. Government supervision inputs tend to be slightly lower than enterprise compliance emission inputs, and low-carbon subsidy is about 40% of enterprise low-carbon investment, so the initial values of both are set to *k* = 50 and *β* = 0.4. Meanwhile, when the governments adopt strict supervision strategy, it forces the enterprises to increase low-carbon investment, at which time the governments obtain additional returns through the increase of industrial production value or the increase of government credibility, which is approximated in this study to be set as *w* = 4. Referring to the study designs of Lu et al. [65] and Li et al. [67] and combining the restrictions on *p* > 0, *α* > 1, 0 < *λ* < 1 and *θ* > 0 in the study assumptions, the initial values of the rest parameters are set as follows: *v* = 45,*p* = 0.2,*α* = 1.5, *θ* = 0.4,*λ* = 0.5. Based on the initial values of the above parameters, this section will dynamically adjust them according to different scenarios, thus revealing the evolution process of the behavioral strategies of the manufacturing enterprises and the governments.

#### Evolutionary trajectory of game system under different scenarios

**Fig 3:**
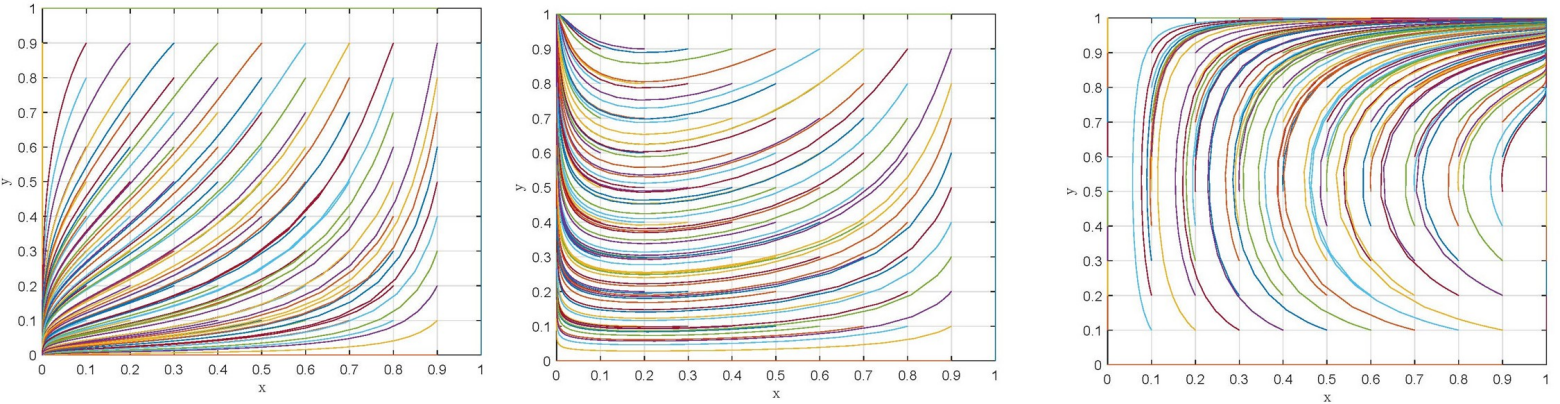
Simulation results of the evolution of game systems under different scenarios. (a) Scenario I. (b) Scenario II. (c) Scenario III.

With the initial assignments of the parameters, the evolutionary trajectories of the game systems under scenarios I and III are simulated, as shown in Fig 3(A) and 3(C), respectively. In addition, taking *λ* = 0.7 and assigning the same values to other parameters, the evolutionary trajectory of the game system under scenario II is obtained, as shown in Fig 3(B). Comparing the evolutionary trajectories of the game systems under the three scenarios, it can be found that: (1) Under the conditions satisfying scenario I and scenario II, *E*_1_(0,0) and *E*_3_(0,1) evolve into ESS after several repeated game, which means that the strategies of supervisory departments are stable in "relaxed supervision" and "strict supervision", respectively, but the strategies of manufacturing enterprises are always stable in "non-compliant emission". (2) Under the condition satisfying scenario III, the game system gradually converges after a short-term oscillation, and the equilibrium point *E*_4_(1,1) evolves to ESS. Consequently, the strategy of supervisory departments is stable in "strict supervision", while the strategy of manufacturing enterprises is stable in "compliant emission".

#### The impact of parameters on players’ behavioral strategy under Model II

From the above simulation results, it can be seen that in the improved Model II, although the game system fails to stabilize at the most ideal equilibrium point *E*_2_(1,0), the "strict supervision" strategy of supervisory departments still drives manufacturing enterprises to prefer the "compliant emission" strategy, that is, the game system stabilizes at an acceptable incentive-compatible state. Therefore, this section further simulates the laws of peer funds (*v*), subsidy coefficient (*β*) and penalty coefficient (*θ*) on the behavioral strategies of manufacturing enterprises and supervisory departments.

**Fig 4:**
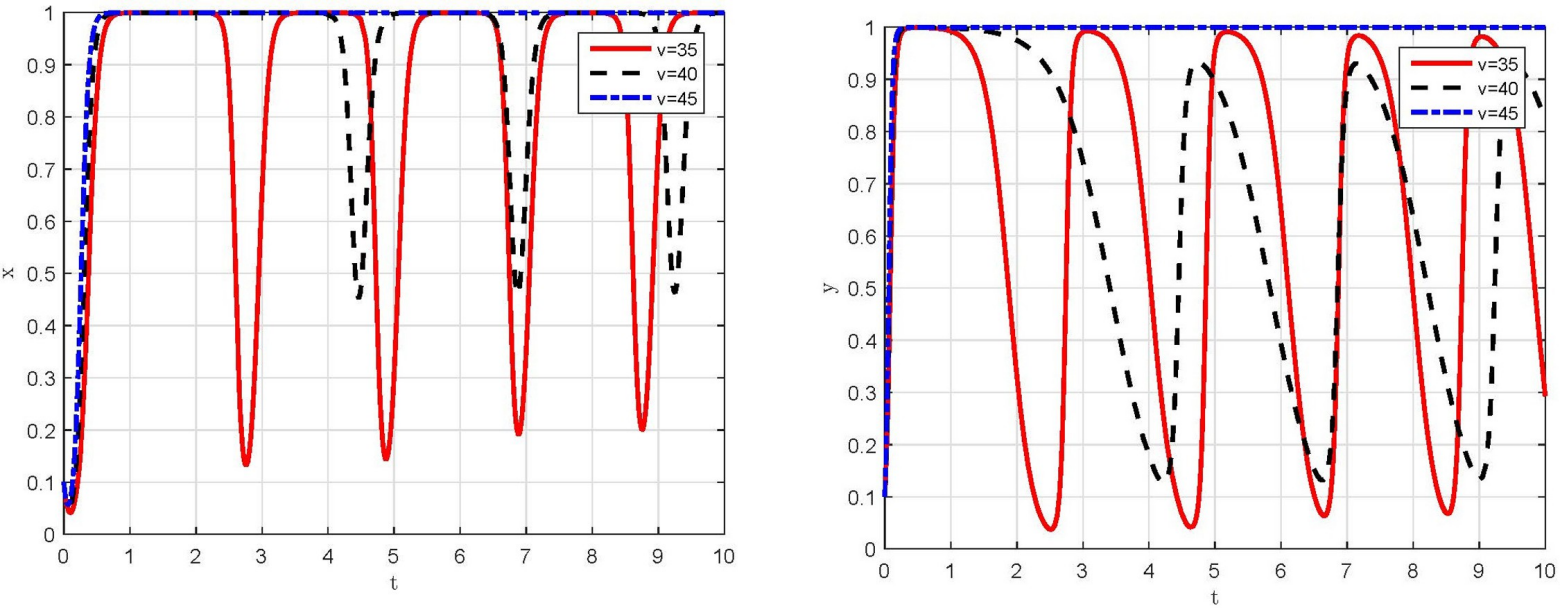
Simulation results of the impact of peer funds on the behavioral strategies of both game players. (a) Manufacturing enterprises. (b) Supervisory departments.

**Fig 5:**
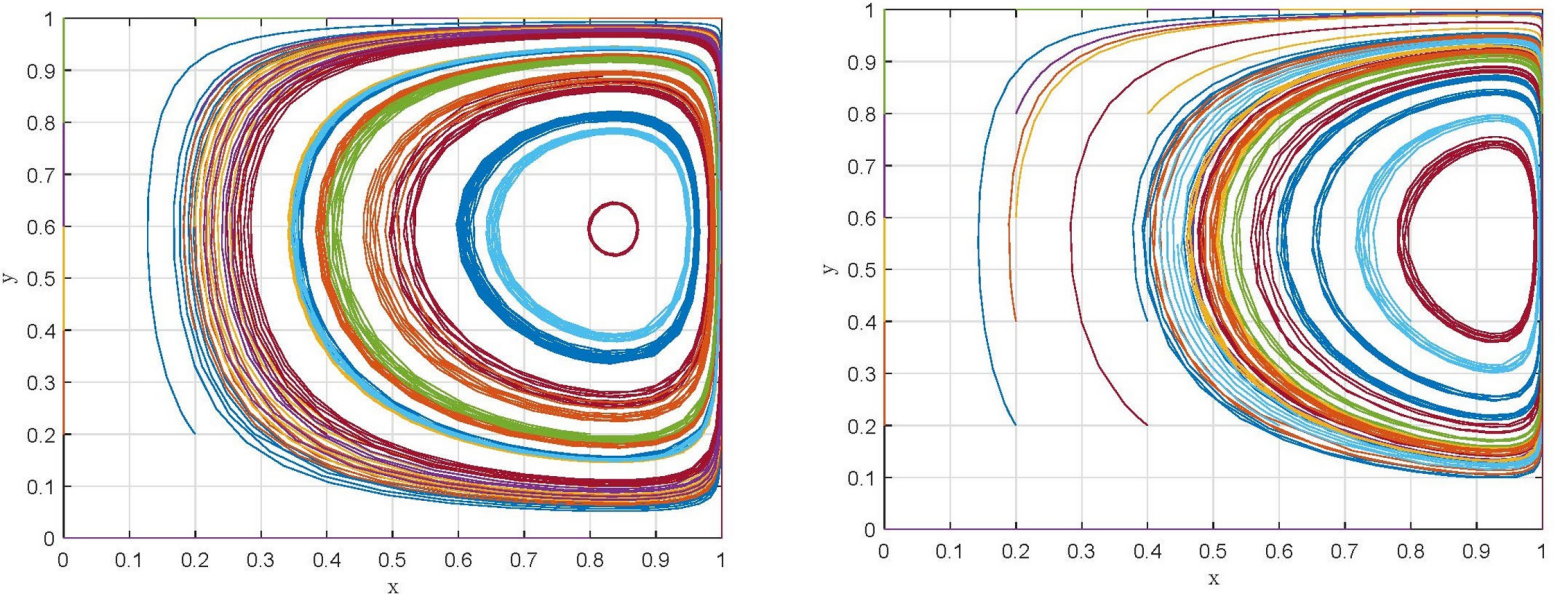
Simulation result of the evolution of the game system under different conditions. (a) *v* = 35. (b) *v* = 40.

*(1) Dynamic evolution in the conditions of parameter v* = 35, *v* = 40 *and v* = 45. Let *v* = 35, *v* = 40 and *v* = 4, respectively, and keep the other parameter assignments the same as the original assignments, the simulation results are shown in Fig 4(A) and 4(B). Under the condition of *v* = 45, the behavioral strategies of manufacturing enterprises and supervisory departments rapidly evolve to "compliant emission" and "strict supervision", which is consistent with the simulation result in Fig 3(C). Moreover, the rate of behavioral strategy evolution to steady state for supervisory departments is faster than that for manufacturing enterprises. Conversely, under the condition of *v* = 35 and *v* = 40, the behavioral strategies of manufacturing enterprises and supervisory departments are unable to evolve to a steady state and lie in a state of dynamic oscillations between different strategies with large range. At this point, the evolutionary trajectory of the game system is shown in Fig 5(A) and 5(B), which present as a closed-loop trajectory with constant oscillatory motion. Manufacturing enterprises and supervisory departments continuously learn and adjust behavioral strategies in accordance with the returns, and always fail to achieve an incentive-compatible state.

**Fig 6:**
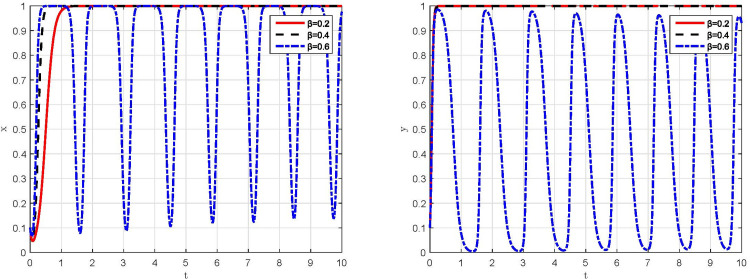
Simulation results of the impact of subsidy coefficient on the behavioral strategies of both game players. (a) Manufacturing enterprises. (b) Supervisory departments.

**Fig 7:**
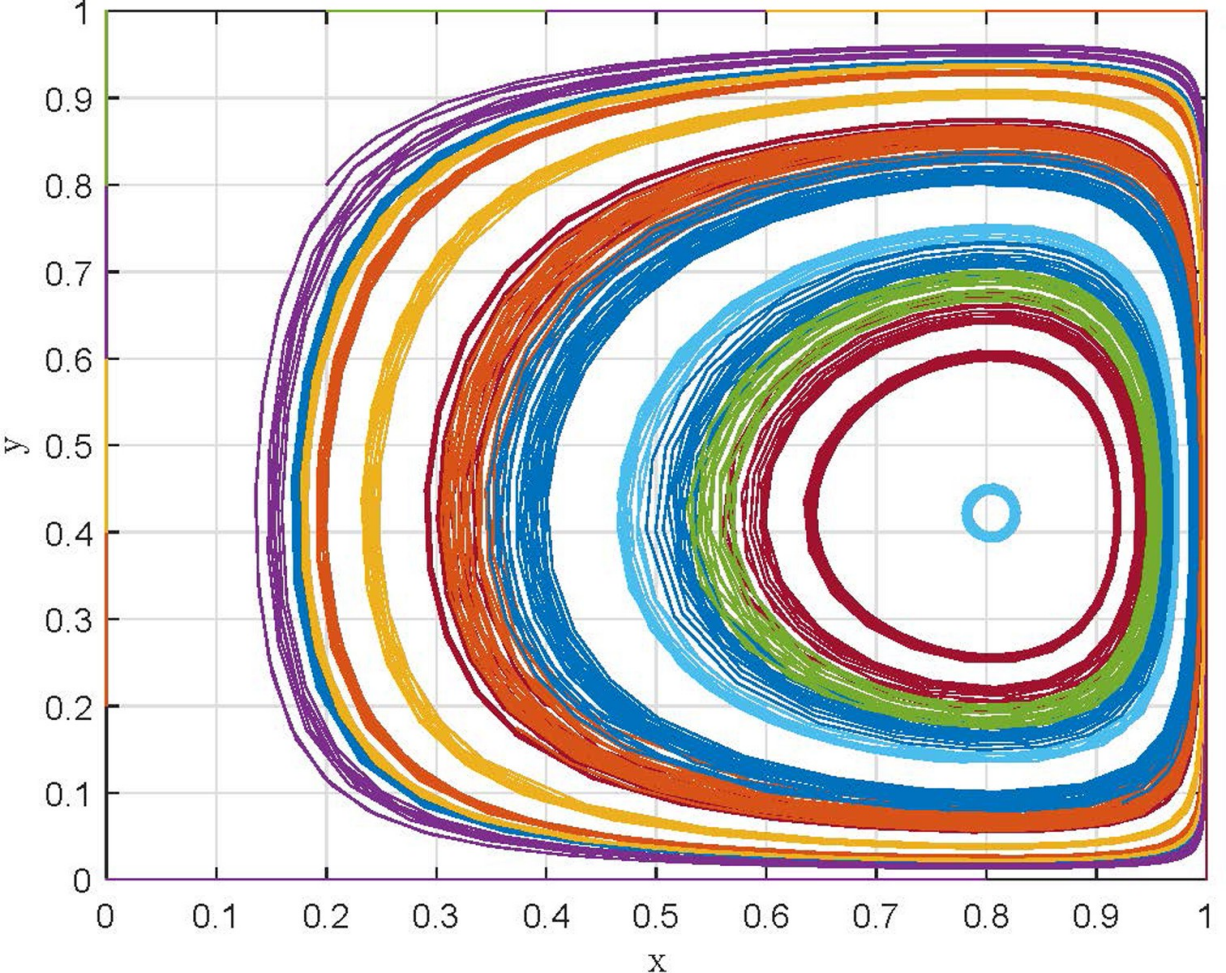
Simulation result of the evolution of the game system under β = 0.6.

*(2) Dynamic evolution in the conditions of parameter β* = 0.2,*β* = 0.4 *and β* = 0.6. Let *β* = 0.2, *β* = 0.4 and *β* = 0.6, respectively, and keep the other parameter assignments the same as the original assignments, the simulation results are shown in Fig 6(A) and 6(B). When *β* = 0.2 or *β* = 0.4, both of which are consistent with the critical range of Scenario III, the behavioral strategies of manufacturing enterprises and supervisory departments evolve to the sub-ideal steady state, respectively, also consistent with the simulation result in Fig 3(C). Meanwhile, with the increase of the subsidy coefficient, the period of behavioral strategy evolution for manufacturing enterprises to "compliant emission" is significantly shorter, while that for supervisory departments to "strict supervision" is not significantly affected. However, when *β* = 0.6, which is beyond the critical range of Scenario III, the behavioral strategies of manufacturing enterprises and supervisory departments present a periodic oscillatory state, and the game system cannot evolve to a stable state, as shown in Fig 7 with a closed-loop trajectory. In other words, when the subsidy coefficient exceeds a critical threshold, the expected returns between manufacturing enterprises and supervisory departments can hardly reach an equilibrium. Ultimately, the choice of behavioral strategies of both sides will enter a vicious cycle of "strict supervision →compliant emission →relaxed supervision → non-compliant emission → strict supervision→ … “.

**Fig 8:**
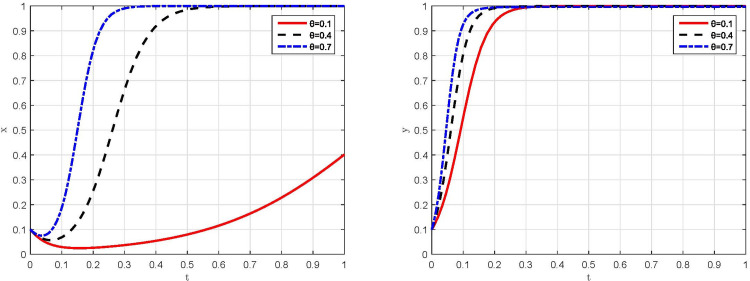
Simulation results of the impact of penalty coefficient on the behavioral strategies of both game players. (a) Manufacturing enterprises. (b) Supervisory departments.

*(3) Dynamic evolution in the conditions of parameter θ =* 0.1,*θ =* 0.4 *and θ =* 0.7. The simulation results are shown in Fig 8(A) and 8(B) under the conditions that θ is taken as 0.1, 0.4 and 0.7, respectively. On the one hand, when *θ* = 0.4 or 0.7, the game system evolves to a steady state of "compliant emission, strict supervision", that is, *E*_4_(1,1) evolves to a stable equilibrium point. With the increase of penalty coefficient, the convergence rate of behavioral strategies to steady state increases significantly for both manufacturing enterprises and supervisory departments, and the increase in the convergence rate of manufacturing enterprises is significantly higher than that of supervisory departments. On the other hand, under the condition of *θ =* 0.1, although the evolution directions of manufacturing enterprises’ and supervisory departments’ behavioral strategy remain the same, but the evolution rate of behavioral strategy for manufacturing enterprises decreases. However, there is a tendency that manufacturing enterprises’ behavioral strategy evolves to "non-compliant emission" for a relatively short period and then to "compliant emission", revealing the oscillatory nature of manufacturing enterprises’ behavioral strategy. Generally, the influence of penalty coefficient on manufacturing enterprises’ behavioral strategy is much greater than that on supervisory departments’ behavioral strategy.

## Results discussion

Due to the existence of information asymmetry in the MCE supervision, manufacturing enterprises are subject to moral hazard and adverse selection, and gain more profits through non-compliant emission. Therefore, effective carbon emission supervision should be characterized by incentive-compatible, which enables manufacturing enterprises to maximize social welfare while pursuing their own profits. For the current incentive mechanism implemented by the Chinese government with high penalties and low rewards, the supervision of supervisory departments over manufacturing enterprises is in an incompatible state. This conclusion supports the argument of Fehr et al. [9] and Herrmann et al. [10] regarding the negative penalty nullification theory in a side way. The main reason is that manufacturing enterprises are risk-neutral rational economic agents, normally maximize economic profits as the behavior criterion, and engage in non-compliant emission behavior depends on the combined value of the expected return and penalty cost. Although the market supervisory departments have taken a variety of punitive measures such as fines, reduction of carbon emission quotas, and exclusion from financing support, it is still difficult to completely curb the violations of manufacturing enterprises to pursue excessive profits. This phenomenon indicates the limitation of the current incentive system and the need to further optimize the supervision mechanism.

When the incentive mechanism with pure penalty is improved to an incentive mechanism with positive subsidy and negative penalty, the supervision of supervisory departments over manufacturing enterprises is changed from an incompatible state to a constrained compatible state. It further highlights the importance of subsidies in incentive-compatible mechanism, which has been demonstrated in the studies of Sun and Chang [7] and Sheng et al. [57]. What needs to be particularly emphasized is that the simulation results fully illustrate the positive effect of peer funds on the compatibility of MCE supervision, which not only expands the funding channels for government subsidies from a practical perspective, but also argues the feasibility of the organizational internal incentive scheme suggested by Yang et al. [68] from a theoretical perspective. However, if the peer funds paid by manufacturing enterprises fail to satisfy a specific threshold, MCE supervision will be in a periodic cycle state of "incentive-compatible → incentive-incompatible → incentive-compatible →…". Following the explanation of Lu et al. [65], insufficient peer funds result that the subsidy funds for MCE supervision mainly come from government finance, and government’s financial pressure may bring intermittent failure of the subsidy effect, which is manifested by the default or suspension of subsidy funds.

## Conclusions

In view of the fact that China’s environmental regulation policies are still evolving, this study applies evolutionary game theory to construct a game model for manufacturing enterprises and supervisory departments, designs an incentive-compatible mechanism that includes two subsidized funding channels of peer funds and government finance. Then, we analyze and compare the compatibility scenarios of MCE supervision under both non-subsidized and subsidized incentives, and simulate the influence laws of peer funds, subsidy coefficient and penalty coefficient on the interactive behavior of manufacturing enterprises and supervisory departments through computational experimental method. The main research conclusions and related recommendations are as follows.

1. Under the non-subsidized incentive mechanism, the MCE supervision is in a state of incentive-incompatible. Corresponding to the reality, a significant portion of local governments in China tend to have difficulty in implementing subsidy policies due to financial pressure, leading to a purely punitive state of MCE supervision. In such a scenario, regardless of the supervisory level or penalty, it is difficult to encourage manufacturing enterprises to choose the ideal strategy of compliant emission, since non-compliant emission can bring greater individual desired returns. With the negative incentive stimulus of pure penalty, CER performance in manufacturing will hardly change substantially, and various carbon violations will still occur. Therefore, it is urgent for supervisory departments to optimize the existing incentive-incompatible mechanism, properly coordinate the potential profit conflicts between Individual manufacturing enterprises and public collectives so as to enhance supervision performance.
2. Under the improved subsidized incentive mechanism, there is a potentially incentive-compatible state for MCE supervision. When the subsidy coefficient satisfies the limit threshold range, the positive subsidy incentive makes manufacturing enterprises and supervisory departments achieve a profit balance under the combination strategy of compliant emission and strict supervision. The incentive system incorporated into the peer funds is consistent with the principle of incentive-compatible, which ensures the profit consistency of manufacturing enterprises and the public. However, in order to prevent fraudulent subsidies, rent-seeking and other problems, it is recommended that supervisory departments should introduce an internal whistle-blower system to encourage internal institutions to report various non-compliant emission behaviors in manufacturing.
3. The incentive-compatible state of MCE supervision has a significant relationship with the peer funds, subsidy coefficient and penalty coefficient. Especially, with the increase of peer funds and penalty coefficient or the decrease of subsidy coefficient, the period of MCE supervision to reach an incentive-compatible state becomes shorter. Conversely, a lower peer fund or a higher subsidy coefficient promotes MCE supervision in a periodic cycle state of "incentive-compatible → incentive-incompatible → incentive-compatible → … ", while a lower penalty coefficient promotes MCE supervision in an incentive-incompatible state. Therefore, under the incentive mechanism of integrating peer funds, financial subsidy and administrative penalty, supervisory departments should moderately increase the peer funds payment and administrative penalty, and accordingly set a reasonable subsidy interval to reduce the opportunistic probability of non-compliant emission behavior of manufacturing enterprises.

This study will be further expanded from two aspects in the future. (1) Some research studies showed that incentives with pure rewards can create more social returns than incentives with penalties [69]. Based on this argument, this study will further compare and analyze the incentive effects of different mechanisms to continuously optimize the incentive-compatible mechanism of MCE supervision. (2) To address the periodic cyclic state of MCE supervision under the static incentive assumption, this study will improve the game model by drawing on the dynamic incentive methods of Lu et al. [65] and Wang et al. [70].

## Supporting information

S1 Appendix Process of model construction. (DOCX)

## References

[1] Sinha RK, Chaturvedi ND. A review on carbon emission reduction in industries and planning emission limits. Renewable and Sustainable Energy Reviews. 2019; 114, 109304. 10.1016/j.rser.2019.109304

[2] Khan AM, Jamil M, Mia M, Zhao W, Gong L. Sustainability based performance evaluation of hybrid nanofluid assisted machining. Journal of Cleaner Production. 2020; 257, 120541. 10.1016/j.jclepro.2020.120541

[3] Avenyo EK, Tregenna F. Greening manufacturing: Technology intensity and carbon dioxide emissions in developing countries. Applied Energy. 2022; 324, 119726. 10.1016/j.apenergy.2022.119726

[4] Deng HH, Zeng QG, Zhao XK. Digital transformation, external pressures and carbon performance in manufacturing companies. Zhejiang Social Sciences. 2023; (10): 36–48. 10.14167/j.zjss.2023.10.012

[5] Lin Q, Zhao ZZ, Huo BF, Lin XG, Li WZ. Retailers’ emissions reduction strategies under carbon trading policy: financing emissions reduction or technological emissions reduction. Systems Engineering-Theory & Practice. 2023; 1–20. https://link.cnki.net/urlid/11.2267.N.20231102.1503.016

[6] An YF, Zhou DQ, Yu J, Shi XP, Wang QW. Carbon emission reduction characteristics for China’s manufacturing firms: Implications for formulating carbon policies. Journal of Environmental Management. 2021; 284, 112055. doi: 10.1016/j.jenvman.2021.112055 33540202

[7] Sun X, Chang Q. Research of the behavior of low-Carbon economy objects based on incentive compatibility theory and game theory. Chinese Journal of Management Science. 2014; 22(S1):794–800. 10.16381/j.cnki.issn1003-207x.2014.s1.078

[8] Jiang K, Zhang X, Wang Y. Stability and influencing factors when designing incentive compatible payments for watershed services: Insights from the Xin’an River Basin. China. Marine Policy. 2021; 134, 104824. 10.1016/j.marpol.2021.104824

[9] Fehr E, Gachter S. Altruistic punishment in humans. Nature. 2002; 415(6868):137–40. doi: 10.1038/415137a 11805825

[10] Herrmann B, Thoni C, Gachter S. Antisocial punishment across societies. Science. 2008; 319(5868):1362–67. doi: 10.1126/science.1153808 18323447

[11] Jin YM., Chen B. Comparison of potential CO_2_ reduction and marginal abatement costs across in the China and Korea manufacturing industries. Journal of Innovation & Knowledge. 2022; 7(2), 100172. 10.1016/j.jik.2022.100172

[12] Li WY, Dong FG, Ji ZG. Evaluation of carbon emission efficiency and reduction potential of 336 cities in China. Journal of Cleaner Production. 2023; 428, 139372. 10.1016/j.jclepro.2023.139372

[13] Zheng J, Shi JJ, Lin F, Hu XY, Pan Q, Qi TN, et al. Reducing manufacturing carbon emissions: Optimal low carbon production strategies respect to product structures and batches. Science of the Total Environment. 2022; 858, 159916. doi: 10.1016/j.scitotenv.2022.159916 36356727

[14] Zhu JH, Lu Y, Song ZT, Shao XF, Yue XG. The choice of green manufacturing modes under carbon tax and carbon quota. Journal of Cleaner Production, 2023; 384, 135336. 10.1016/j.jclepro.2022.135336

[15] Ma Y, Li FY, Wang LM, Wang G. Multidimensional evaluation method and application based on life cycle carbon efficiency considering carbon emission, cost, and fuction. Environmental Science and Pollution Research. 2023; 30(27): 70918–36. 10.1007/s11356-023-27290-w 37156949

[16] Guo X, Chen L, Wang J, Liao LH. The impact of disposability characteristics on carbon efficiency from a potential emissions reduction perspective. Journal of Cleaner Production. 2023; 408, 137180. 10.1016/j.jclepro.2023.137180

[17] Ke Y, Liu J, Meng J, Fang SN, Zhuang SQ. Comprehensive evaluation for plan selection of urban integrated energy systems: A novel multi-criteria decision-making framework. Sustainable Cities and Society. 2022; 81, 103837. 10.1016/j.scs.2022.103837

[18] Mahapatra B, Irfan M. Asymmetric impacts of energy efficiency on carbon emissions: a comparative analysis between developed and developing economies. Energy. 2021; 227, 120485. 10.1016/j.energy.2021.120485

[19] Lei W, Xie Y, Hafeez M, Ullah S. Assessing the dynamic linkage between energy efficiency, renewable energy consumption, and CO_2_ emissions in China. Environmental Science and Pollution Research. 2022; 29, 19540–52. 10.1007/s11356-021-17145-7 34718974

[20] Dong F, Qin C, Zhang XY, Zhao X. Towards carbon neutrality: The impact of renewable energy development on carbon emission efficiency. International Journal of Environmental Research and Public Health. 2021; 18(24), 13284. doi: 10.3390/ijerph182413284 34948893 PMC8701276

[21] Li FY, Cao X, Sheng PP. Impact of pollution-related punitive measures on the adoption of cleaner production technology: Simulation based on an evolutionary game model. Journal of Cleaner Production. 2022; 339, 130703. 10.1016/j.jclepro.2022.130703

[22] Jin B, Han Y, Kou P. Dynamically evaluating the comprehensive efficiency of technological innovation and low-carbon economy in China’s industrial sectors. Socio-Economic Planning Sciences. 2023; 86, 101480. 10.1016/j.seps.2022.101480

[23] Zhu JH, Feng TW, Lu Y, Jiang WB. Using blockchain or not? A focal firm’s blockchain strategy in the context of carbon emission reduction technology innovation. Business Strategy and the Environment, 2024; Vol ahead-of-print. 10.1002/bse.3664

[24] Jain R, Mittal M, Mangla SK, Baraiya R. Optimizing supply chain strategies for deteriorating items and imperfect manufacturing under carbon emission regulations. Computers & Industrial Engineering. 2023; 182, 109350. 10.1016/j.cie.2023.109350

[25] Mondal C, Giri BC. Retailers’ competition and cooperation in a closed-loop green supply chain under governmental intervention and cap-and-trade policy. Operational Research. 2022; 22: 859–94. 10.1007/s12351-020-00596-0

[26] Khan Y, Oubaih H, Elgourrami FZ. The effect of renewable energy sources on carbon dioxide emissions: evaluating the role of governance, and ICT in Morocco. Renew. Energy. 2022; 190: 752–763. 10.1016/j.renene.2022.03.140

[27] Murshed M, Apergis N, Alam MS, Khan U, Mahmud S. The impacts of renewable energy, financial inclusivity, globalization, economic growth, and urbanization on carbon productivity: Evidence from net moderation and mediation effects of energy efficiency gains. Renewable Energy. 2022; 196: 824–38. 10.1016/j.renene.2022.07.012

[28] Bhandari D, Singh RK, Garg SK. Prioritisation and evaluation of barriers intensity for implementation of cleaner technologies: Framework for sustainable production. Resources, Conservation and Recycling. 2019; 146: 156–67. 10.1016/j.resconrec.2019.02.038

[29] An YF, Shi XP, Wang QW, Yu J, Zhou DQ, Zhou XY. China’s manufacturing firms’ willingness to pay for carbon abatement: A cost perspective. Business Strategy and the Environment. 2023. 10.1002/bse.3431

[30] Wu K, Bai E, Zhu HJ, Lu ZJ, Zhu HX. A tripartite evolutionary game behavior analysis of the implementation strategy of the internal carbon pricing of enterprises under governments supervision. Heliyon, 2023; 9(12), e23131. doi: 10.1016/j.heliyon.2023.e23131 38144269 PMC10746489

[31] Wang J. The Theoretical system of government regulation with Chinese characteristics: Demand analysis, constructing orientations and overall framework. Journal of Management World. 2021; 37(2): 148–164+184+11. 10.19744/j.cnki.11-1235/f.2021.0025

[32] Yuan KH, Cui JY, Zhang HP, Gao X. Do cleaner production standards upgrade the global value chain position of manufacturing enterprises? Empirical evidence from China. Energy Economics. 2023; 128,107185. 10.1016/j.eneco.2023.107185

[33] Tang K, Qiu Y, Zhou D. Does command-and-control regulation promote green innovation performance? Evidence from China’s industrial enterprises. Science of The Total Environment. 2020; 712, 136362. doi: 10.1016/j.scitotenv.2019.136362 31935549

[34] Xiong H, Jing Z, Zhan J, Zhan JT. Impact of different environmental regulatory tools on technological innovation of Chinese industrial enterprises above designated size. Resource Science. 2020; 42: 1348–60. 10.18402/resci.2020.07.11

[35] Liu L, Li MY, Gong XJ, Jiang P, Jin RF, Zhang YH. Influence mechanism of different environmental regulations on carbon emission efficiency. International Journal of Environmental Research and Public Health. 2022; 19, 13385. doi: 10.3390/ijerph192013385 36293964 PMC9602758

[36] Perera K, Kuruppuarachchi D, Kumarasinghe S, Suleman MT. The impact of carbon disclosure and carbon emissions intensity on firms’ idiosyncratic volatility. Energy Economics. 2023; 128, 107053. 10.1016/j.eneco.2023.107053

[37] Perera L, Jubb C, Gopalan S. A comparison of voluntary and mandated climate change-related disclosure. Journal of Contemporary Accounting&Economics.2019;15(2): 243–66. 10.1016/j.jcae.2019.100157

[38] Klassen RD, Shafiq A, Johnson F. Opportunism in supply chains: Dynamically building governance mechanisms to address sustainability-related challenges. Transportation Research Part E: Logistics and Transportation Review. 2023; 171, 103021. 10.1016/j.tre.2023.103021

[39] Chen H, Hao Y, Li J, Song X. The impact of environmental regulation, shadow economy, and corruption on environmental quality: theory and empirical evidence from China. Journal of Cleaner Production. 2018; 195: 200–214. 10.1016/j.jclepro.2018.05.206

[40] Peng P. Study on legal liability assumption under the third-party environmental pollution control model. Environment and Sustainable Development. 2019; 44(4): 94–99. 10.19758/j.cnki.issn1673-288x.201904094

[41] Yuan HX, Zou LH, Feng YD. How to achieve emission reduction without hindering economic growth? The role of judicial quality. Ecological Economics. 2023; 209, 107839. 10.1016/j.ecolecon.2023.107839

[42] Meng LP, Liu KM, He JL, Han CF, Liu PH. Carbon emission reduction behavior strategies in the shipping industry under government regulation: A tripartite evolutionary game analysis. Journal of Cleaner Production. 2022; 378, 134556. 10.1016/j.jclepro.2022.134556

[43] Jiang K, Zhang LL, Zhang XJ, Wang YS. Sustainable implementation of the carbon-labeling policy with customer participation and government supervision. Computers & Industrial Engineering. 2023; 178, 109100. 10.1016/j.cie.2023.109100

[44] Kane EJ. Principal-agent problems in S&L salvage. The Journal of Finance. 1990; 45(3): 755–64. 10.1111/j.1540-6261.1990.tb05104.x

[45] You JS, Juarez R. Incentive-compatible simple mechanisms. Economic Theory. 2021; 71, 1569–89. 10.1007/s00199-021-01342-z

[46] Gibbard A. Manipulation of voting schemes: A general result. Econometrica. 1973; 41(4): 587–601. 10.2307/1914083

[47] Satterthwaite MA. Strategy-proofness and arrow’s conditions: Existence and correspondence theorems for voting procedures and social welfare functions. Journal of Economic Theory. 1975; 10(2): 187–217. 10.1016/0022-0531

[48] David N. Efficient renewable electricity support: Designing an incentive-compatible support scheme. Energy Journal. 2023; 44(3): 1–22. 10.5547/01956574.44.3.dnew

[49] Xie HP, Wang Y, Chen C, Bie ZH. Pricing for TSO-DSO coordination: A decentralized incentive compatible approach. IEEE Transactions on Power Systems. 2023; 38(2):1867–80. 10.1109/TPWRS.2022.3170436

[50] Enaleev AK. Optimal incentive compatible mechanism in a system with several active elements. Automation and Remote Control. 2017; 78(1): 146–158. 10.1134/S000511791701012X

[51] Mansour Y, Slivkins A, Syrgkanis V. Bayesian Incentive-compatible bandit exploration. Operations Research. 2020; 68(4): 1132–61. 10.1287/opre.2019.1949

[52] Jiang M, Guo QL, Sun HB, Ge HC. Leverage reactive power ancillary service under high penetration of renewable energies: An Incentive-compatible obligation-based market mechanism. IEEE Transactions on Power Systems. 2022; 37(4): 2919–33. 10.1109/TPWRS.2021.3125093

[53] Böhringera C, Rutherfordb TF, Springmann M. Clean-Development investments: An incentive-compatible CGE modeling framework. Environmental & Resource Economics. 2015; 60(4): 633–51. 10.1007/s10640-014-9762-3

[54] Kunimoto T, Zhang C. On incentive compatible, individually rational public good provision mechanisms. Social Choice and Welfare. 2021; 57(2): 431–68. 10.1007/s00355-021-01329-8

[55] Zhu JH, Baker JS, Song ZT, Yue XG, Li WQ. Government regulatory policies for digital transformation in small and medium-sized manufacturing enterprises: an evolutionary game analysis. Humanities & Social Sciences Communications. 2023; 10, 751, 10.1057/s41599-023-02250-4

[56] Dindo P, Tuinstra J. A class of evolutionary models for participation games with negative feedback. Computational Economics. 2011; 37: 267–300. 10.1007/s10614-011-9253-3

[57] Sheng J, Webber M. Incentive-compatible payments for watershed services along the Eastern Route of China’s South-North Water Transfer Project. Ecosystem Services. 2017; 25: 213–26. 10.1016/j.ecoser.2017.04.006

[58] Sheng J, Zhou W, Zhu B. The coordination of stakeholder interests in environmental regulation: Lessons from China’s environmental regulation policies from the perspective of the evolutionary game theory. Journal of Cleaner Production. 2020; 249, 119385. 10.1016/j.jclepro.2019.119385

[59] Wu SJ, Sun XY, Yang P. Game analysis of carbon emission regulation under dual governance system. China population, resources and environment. 2017; 27(12): 21–30. 10.12062/cpre.20170705

[60] Smith JM. The theory of games and the evolution of animal conflicts. Journal of theoretical biology. 1974; 47(1): 209–21. doi: 10.1016/0022-5193(74)90110-6 4459582

[61] Wei M, Zhao Y, Xia Y. The evolution of online P2P lending risk: Game based on platform and supervisor. Management Review. 2021; 33(3): 54–65. 10.14120/j.cnki.cn11-5057/f.2021.03.004

[62] Horlick-Jones T. Informal logics of risk: contingency and modes of practical reasoning. Journal of Risk Research. 2005; 8(3): 253–72. 10.1080/1366987042000270735

[63] Liu JG, Wang JJ, Zhou H, Zhang YQ. Research on supervision problems of port hazardous chemicals base on security risk level. Systems Engineering-Theory & Practice. 2018; 38(5): 1141–52. 10.12011/1000-6788(2018)05-1141-12

[64] Friedman D. On economic applications of evolutionary game theory. Journal of Evolutionary Economics. 1998; 8(1): 15–42. 10.1007/s001910050054

[65] Lu C, Cheng H, Cai J. Evolutionary game analysis: Impacts of government subsidies on manufacturers’ green R&D under peer incentive mechanism. Chinese Journal of Management. 2022; 19(1): 96–101. 10.3969/j.issn.1672-884x.2022.01.011

[66] Fang G, He Y, Tian L. Evolutionary game analysis of government and enterprises carbon-reduction under the driven of carbon trading. Chinese Journal of Management Science. 2021. 10.16381/j.cnki.issn1003-207x.2021.1401

[67] Li S, Cai J, Feng Z, Xu YF, Cai HB. Government contracting with monopoly in infrastructure provision: Regulation or deregulation? Transportation Research Part E: Logistics and Transportation Review. 2019; 122: 506–523. 10.1016/j.tre.2019.01.002

[68] Yang CL, Zhang B, Charness G, Li C, Lien JW. Endogenous rewards promote cooperation. Proceedings of the National Academy of Sciences of the United States of America. 2018; 115(40): 9968–73. doi: 10.1073/pnas.1808241115 30224497 PMC6176598

[69] Rand DG, Dreber A, Ellingsen T, Fudenberg D, Nowak MA. Positive interactions promote public cooperation. Science. 2009; 325(5945): 1272–75. doi: 10.1126/science.1177418 19729661 PMC2875121

[70] Wang CY, Cui WF. Supervision for the public health services for older adults under the background of government purchasing: An evolutionary game analysis framework. Frontiers in Public Health. 2022; 10, 881330. doi: 10.3389/fpubh.2022.881330 35651859 PMC9149156

