# Supplementary material for: Incentive-compatible mechanism for manufacturing carbon emission supervision under carbon control policies in China
Source: PLoS One. 2024 May 13;19(5):e0299086. doi: 10.1371/journal.pone.0299086 (PMC11090604; doi:10.1371/journal.pone.0299086)
Supplement: S1 Appendix — (DOCX) [file pone.0299086.s001.docx]

**Appendix. Process of model construction**

According to the Table 2, the expected return$U_{11}$for the "compliant emission" strategy, the expected return$U_{12}$for the "non-compliant emission" strategy, and the average expected return$\bar{U}_{1}$can be obtained, respectively.

$$\left\{ \begin{matrix} U_{11}=y\left( r-c \right)+\left( 1-y \right)\left( r-ac \right) \\ U_{12}=y\left[ r-c-\theta\alpha^{2}(\alpha-1)c \right]+(1-y)(r-c) \\ \bar{U}_{1}=xU_{11}+\left( 1-x \right)U_{12} \end{matrix} \right.$$

Similarly, the expected return$U_{21}$for the "strict supervision" strategy, the expected return$U_{22}$for the "relaxed supervision" strategy, and the average expected return $\bar{U}_{2}$can be obtained, respectively.

$$\left\{ \begin{matrix} U_{21}=x\left( w-k \right)+\left( 1-x \right)[w-k+\theta\alpha^{2}\left( \alpha-1 \right)c] \\ U_{22}=x\left( w-\lambda k \right)+\left( 1-x \right)\left[ \left( 1-p \right)w-\lambda k \right] \\ \bar{U}_{2}=yU_{21}+\left( 1-y \right)U_{22} \end{matrix} \right.$$

Then, the replication dynamic equations of manufacturing enterprises and supervisory departments can be calculated, respectively.

$$F\left( x \right)=x\left( U_{11}-\bar{U}_{1} \right)$$

$$F\left( y \right)=y\left( U_{21}-\bar{U}_{2} \right)$$
